# Motor Asymmetry Attenuation in Older Adults during Imagined Arm Movements

**DOI:** 10.3389/fnagi.2014.00049

**Published:** 2014-03-20

**Authors:** Christos Paizis, Xanthi Skoura, Pascaline Personnier, Charalambos Papaxanthis

**Affiliations:** ^1^Unité de Formation et de Recherche en Sciences et Techniques des Activités Physiques et Sportives, Université de Bourgogne, Dijon, France; ^2^Unité 1093, Cognition, Action et Plasticité Sensorimotrice, Institut National de la Santé et de la Recherche Médicale, Dijon, France; ^3^Sport Science Faculty, Center for Performance Expertise G. Cometti, University of Burgundy, Dijon, France

**Keywords:** aging, motor asymmetry, motor imagery, movement duration, right arm, left arm

## Abstract

Laterality is an important feature of motor behavior. Several studies have shown that lateralization in right-handed young adults (i.e., right versus left arm superiority) emerges also during imagined actions, that is when an action is internally simulated without any motor output. Such information, however, is lacking for elderly people and it could be valuable to further comprehend the evolution of mental states of action in normal aging. Here, we evaluated the influence of age on motor laterality during mental actions. Twenty-four young (mean age: 24.7 ± 4.4 years) and 24 elderly (mean age: 72.4 ± 3.6 years) participants mentally simulated and actually executed pointing movements with either their dominant-right or non-dominant-left arm in the horizontal plane. We recorded and analyzed the time of actual and mental movements and looked for differences between groups and arms. In addition, electromyographic activity from arm muscle was recorded to quantify any enhancement in muscle activation during mental actions. Our findings indicated that both groups mentally simulated arm movements without activating the muscles of the right or the left arm above the baseline level. This finding suggests that young and, notably, elderly adults are able to generate covert actions without any motor output. We found that manual asymmetries (i.e., faster movements with the right arm) were preserved in young adults for both actual and mental movements. In elderly adults, manual asymmetries were observed for actual but not for mental movements (i.e., equal movement times for both arms). These findings clearly indicate an age-related reduction of motor laterality during mental actions.

## Introduction

During imagined actions subjects internally simulated a movement from a first person perspective (i.e., kinesthetic movement representation) without any motor output. Several neuroimaging studies have shown that almost the same neural network is involved in mental movement simulation and actual movement production (Decety, 1996; Jeannerod, 2001; Guillot and Collet, 2005; Munzert and Zentgraf, 2009; Munzert et al., 2009). Specifically, the posterior parietal cortex, the premotor cortex, the supplementary motor area, the primary motor cortex, the basal ganglia, and the cerebellum are activated during overt and covert states of action. At the physiological level, the activation of the autonomic nervous system increases proportionally to the mental effort produced during mental movements (Decety et al., 1993; Demougeot et al., 2009; Collet et al., 2013). Furthermore, motor imagery training enhances muscular force (Yue and Cole, 1992; Zijdewind et al., 2003; Ranganathan et al., 2004) and improves motor performance (Yaguez et al., 1998; Gentili et al., 2006, 2010; Wohldmann et al., 2007; Allami et al., 2008; Louis et al., 2008; Avanzino et al., 2009). Finally, psychophysical investigations have demonstrated that movement execution and its mental replication follow the same laws of movement control (Decety et al., 1989; Decety and Jeannerod, 1995; Maruff et al., 1999b; Bakker et al., 2007; Gueugneau et al., 2008; Gueugneau and Papaxanthis, 2010; Papaxanthis et al., 2012).

It has been postulated that forward internal models are in the core of mental states of action and provide an interesting theoretical basis for understanding their behavioral similarities with motor states. Forward models mimic the causal flow of the physical process by predicting the consequences (e.g., position, velocity) of a motor command (Wolpert and Miall, 1996; Wolpert and Flanagan, 2001; Demougeot and Papaxanthis, 2011). During mental actions, although no overt movement occurs, a corollary discharge (i.e., efference copy of the motor command) and the current state of the body are both available to the forward model which provides future state estimations. State prediction can thus be used to monitor whether an ongoing movement proceeds as planned. It has been proposed that the involvement of the same internal models in actual movement production and mental movement simulation may explain the tight temporal similarities between actual and mental movements (Gueugneau et al., 2009; Demougeot and Papaxanthis, 2011; Papaxanthis et al., 2012).

It is important to note that vividness and accuracy of mental actions decline in later life (Skoura et al., 2005, 2008; Malouin et al., 2007; Mulder, 2007; Saimpont et al., 2009; Personnier et al., 2010a,b). For instance, it has been shown that elderly people did not follow Fitts’s law during imagined arm movements (Skoura et al., 2008) and that the estimation of hand orientation (i.e., mental rotation paradigm) is severely weakened in aged adults (Saimpont et al., 2009). Fitts’s law postulates that the time required to rapidly move to a target area is a function of the distance to the target and the size of the target (Fitts, 1954). An intruding question is whether age-related changes in motor imagery ability concern equally the dominant-right and the non-dominant-left arm. Previous studies involving young adults have shown that lateralization (i.e., right versus left arm superiority) in right-handers also emerged in imagined movements (Maruff et al., 1999a; Skoura et al., 2008, 2009). Such information, however, is lacking for elderly people and it could be valuable to further comprehend the evolution of mental states of action in normal aging.

A careful inspection of the literature reveals contradictory findings regarding manual asymmetries in elderly adults. One the one hand, it has been shown that significant neural adaptations in the corticospinal control of the non-dominant-left arm occur with age (Hutchinson et al., 2002; Sale and Semmler, 2005). For instance, lower motor evoked potentials and shorter silent-period durations in the non-dominant-left hand have been reported when comparing elderly with young adults, whereas there was no age difference in the dominant-right hand (Sale and Semmler, 2005). Psychophysical studies have also shown that the non-dominant-left hand is more affected than the dominant-right hand (Francis and Spirduso, 2000; Personnier et al., 2008; Skoura et al., 2008; Saimpont et al., 2009). According to this literature, one should expect manual asymmetries (i.e., right arm superiority in right-handers) to increase with age in mental actions. Such an outcome could also be expected from the *right hemi-aging hypothesis*, which postulates that age-related cognitive decline affects functions attributed to the right hemisphere to a greater degree than those associated with the left hemisphere (Brown and Jaffe, 1975). As mental movements are lateralized in the left motor cortex of right-handers (Fadiga et al., 1999; Sabate et al., 2004; Stinear et al., 2006), one could anticipate an increase in the right arm dominance in later life. On the other hand, hemispheric recruitment becomes more symmetric (Reuter-Lorenz and Lustig, 2005; Seidler et al., 2010) and manual asymmetry decreases with age (Kalisch et al., 2006; Przybyla et al., 2011). In this line, the *HAROLD* model proposes that frontal activity during cognitive performance tends to be less lateralized in older than in younger adults (Cabeza, 2002). In addition, older adults recruited additional cortical and subcortical areas compared to young adults for the performance of motor tasks (Mattay et al., 2002; Heuninckx et al., 2005, 2008). This strategic difference in the older adults’ brain activation was associated with increased motor performance, a finding which supports compensation over dedifferentiation processes (Heuninckx et al., 2008). According to these findings, it seems reasonable to anticipate a reduction in manual asymmetry during mental actions.

Here, we tested these two alternative hypotheses by exploring the timing features of actual and imagined arm movements in healthy young and aged adults. Precisely, right-handed participants carried out actual and mental movements with their dominant-right and non-dominant-left arms between three targets in the frontal plane. We analyzed the time of actual and mental movements and looked for differences between arms and groups. Our findings clearly showed an age-related attenuation of laterality during mental actions.

## Materials and Methods

### Participants

Forty-eight right-handed volunteers participated in the present study. They were divided into two different groups according to their age: the young group (11 males and 13 females; mean age: 24.7 ± 4.4 years) and the elderly group (10 males and 14 females; mean age: 72.4 ± 3.6 years). Young participants were students from the Université de Bourgogne. Elderly participants were all retired, did regular physical activity (~1.5 h 2 days/week approved by a medical doctor), and at least one daily cognitive activity (reading newspapers, books, or crosswords). Elderly and young participants were in good health, with normal or corrected vision and without any nervous, muscular, or cognitive disorders. None of them had a medical treatment for a chronic pathology. Elderly participants were recruited from fitness club. Right arm dominance was determined by means of the *Edinburgh Handedness inventory* (Oldfield, 1971). Handedness scores were not different between the two groups [on average, young = 0.87 ± 0.04 and elderly = 0.86 ± 0.04; *t* = 0.98, degree of freedom df = 46, *P* = 0.33]. Participants received complete information about the experimental procedures but not of the aim of the experiment. Informed consents were signed by all participants and the regional ethics committee of Burgundy (CER) approved the experimental protocol which was carried out in agreement with legal requirements and international norms (Declaration of Helsinki, 1964).

### Experimental protocol

**Figure 1:**
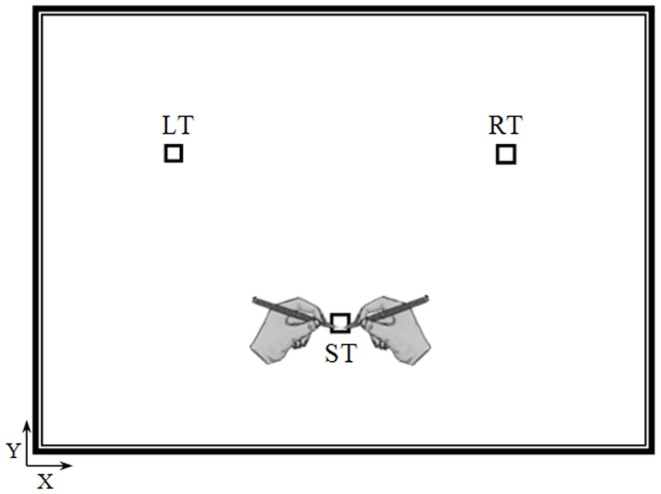
**Top view of the experimental device**. Elderly and young participants performed actual and mental pointing movements between the three targets with their right or left arm. The distance and the angle between the starting target (ST) and the other two targets (the right target, RT and the left target, LT) were 20 cm and 90°, respectively.

The experiment took place in a sound-attenuated room. Participants were seated on an armless and adjustable chair in front of a horizontal panel (see Figure 1) in which we drew three targets (squares, 1 cm × 1 cm): the starting target (ST), the right target (RT), and the left target (LT). The distance and the angle between the ST and the other two targets (RT and LT) were 20 cm and 90°, respectively. The position of the targets on the panel was adapted to each participant’s height, so that the ST was located at the center of the participant’s chest (i.e., the middle of the line connecting the right and left shoulder), which was parallel to the horizontal panel at a distance of 35 cm.

Young and elderly adults had to point (actual movements) or to imagine pointing (mental movements) between the targets very *accurately and as fast as possible*, while holding a pencil either with their dominant-right or non-dominant-left arm. For the mental trials, we emphasized to them that they must feel themselves performing the task (motor or internal imagery (as reported by Personnier et al., 2010b) rather than visualizing themselves doing it (visual or external imagery). Imagining a movement in the first person is a necessary condition to engage the motor system (Stinear et al., 2006). It is known that relatively long trial durations are necessary to obtain reliable measurements in motor imagery protocols because movement durations have a coarse resolution (Sirigu et al., 1996; Papaxanthis et al., 2012). Therefore, in our protocol one actual or one mental trial consisted of eight successive and alternate pointing movements between the three targets. The trial always started from the ST and the first pointing movement was oriented either to the RT (dominant-right arm) or to the LT (non-dominant-left arm) followed by seven other pointing movements; for example: ST, RT, ST, LT, ST, RT, ST, LT (dominant-right arm), and ST or ST, LT, ST, RT, ST, LT, ST, RT, and ST (non-dominant-left arm). During mental trials, participants maintained the operating arm motionless at the starting position. No specific instructions were provided regarding eye movements, but participants were instructed to avoid head movements.

Prior to the experiment and after receiving a demonstration by the experimenter, all participants actually practiced three times. The results of these trials were not included in the main experiment. All participants reported having understood the task requirements and none of them had expressed any inconvenience regarding their actual performance. Mental practice trials were also performed. After having practiced 6–10 times, all participants reported being able to imagine the movement in a first person perspective. Participants carried out 10 actual and 10 mental trials for each operating arm (a total of 40 trials). Movement (actual and mental) and arm (dominant-right and non-dominant-left) conditions were presented in a block design (four sessions) and were counterbalanced between the participants. Trials were separated by a time-interval of at least 5 s and sessions by a time-interval of 5 min. The whole experiment lasted ~40 min/participant.

### Spatial accuracy of actual movements

For each actual movement, we controlled final precision by verifying that each mark made by the pencil was inside or outside the target. Participants were informed that if they missed more than two targets during an actual movement, this trial would not be considered and would be performed again. Spatial accuracy between elderly and young participants was assessed by measuring the number of invalid trials (i.e., the trials during which a participant missed more than two targets). Following this restriction, a small number of trials were repeated. Precisely, for the right arm, 9 trials out of 240 (24 participants × 10 trials) were repeated by the young group (3.8%) and 11 trials (4.6%) by the elderly group. Regarding the left arm, eight trials were repeated by the young and the elderly group (3.3%). Spatial accuracy was equivalent between groups for actual movements (for both arms, *t* < 1, df = 46, *P* > 0.80) and thus permitted the comparison of actual movement durations between groups and arms. Spatial accuracy cannot be measured during mental movements, since no overt movement occurs. For that reason, we explicitly asked participants to respect our requirements; that is, to make mental movements as accurate and as fast as possible.

### Data recording

Actual and mental trials were recorded using the Biopac MP150 (Biopac System, CA, USA) and an electronic stopwatch (temporal resolution 1 ms). The Biopac served for the recording of muscle activation patterns during mental trials and the electronic stopwatch for the recording of movement duration. Before an actual or a mental trial, participants placed the pencil with their right or left arm on the center of the ST and remained motionless. They also held the electronic stopwatch in their left hand (when pointing with the right arm) or right hand (when pointing with the left arm). After a variable temporal interval (between 2 and 3 s), the experimenter started the trial acquisition. After 1 s (S1), an auditory cue was given as the starting signal for each actual or mental trial. After this auditory cue, participants started the stopwatch when they physically or mentally initiated the movement and they stopped it when they had physically or mentally completed it (at the same instance the experimenter also stopped the trial acquisition). We required participants to record their actual and mental movement durations because they reported that they felt more comfortable (especially the elderly participants) manipulating the stopwatch themselves. We had previously validated this method in both young and elderly subjects (Papaxanthis et al., 2003; Skoura et al., 2005; Gueugneau et al., 2008; Personnier et al., 2010b). We evaluated the level of muscle activation during mental movements by recording EMG signals from two flexor muscles, the anterior deltoid (AD, flexor of the shoulder joint), and the biceps brachii (BB, flexor of the shoulder and the elbow joints), and from two extensor muscles, the posterior deltoid (PD, extensor of the shoulder joint), and the triceps brachii (TB, extensor of the shoulder and the elbow joints). Two silver-chloride surface electrodes of 10 mm diameter were positioned on the muscle belly (with the skin previously shaved and cleaned) with an inter-electrode distance (center–center) of 2 cm. The reference electrode was placed on the left ankle. EMG signals were recorded at a frequency of 1000 Hz, filtered with a zero-phase lag fifth order Butterworth filter with 20–400 Hz band pass cut-off frequency, and stored for off-line analysis using MATLAB^®^ software acquisition.

### EMG patterns

In the EMG analysis, we used: (i) the 1-s temporal interval (S1), that from the beginning of movement recording until the auditory cue, as the reference period for the calculation of the EMG baseline level (BL), and (ii) the temporal interval between the auditory cue and the end of movement recording as the movement period for the quantification of the level of muscle activation during mental trials. We analyzed the EMG activity of all mental trials within the temporal window between 10 and 90% of the whole movement acquisition (as reported by Personnier et al., 2010a). In this way, we were certain that participants had already started, but still not finished their mental trials.

We quantitatively analyzed the EMG patterns of the muscles during mental trials by computing their activation level (root mean square, RMS) using the following formula:

$$\text{RMS}=\sqrt{\frac{1}{\text{MD}}\int_{0}^{\text{MD}}{\text{(EMG)}}^{2}\text{dt}}$$

where MD represents the movement duration.

We calculated the RMS values of the baseline (RMS_BL_) and the RMS values during mental movements (RMS_M_) for each participant. We verified that all variables showed normal distribution (Shapiro–Wilk test, *P* > 0.05) and then we compared the RMS_M_ with the RMS_BL_ by means of paired *t*-tests for each group, operating arm, and muscle separately.

### Data and statistical analysis

#### Movement duration

For each participant, we calculated the average movement duration in each experimental condition. Variables showed normal distribution (Shapiro–Wilk test, *P* > 0.05) and equivalent variance (Levene test, *P* > 0.05). Therefore, we performed an analysis of variance (ANOVA) with *age* (young-elderly) as between subject factor and *arm* (right-left), *movement* (actual, mental) as within-subject factors. *Post hoc* differences were assessed by means of *Tukey* tests and the level of significance was fixed at *P* < 0.05.

#### Manual asymmetry

To evaluate the degree of manual asymmetry in actual and mental movements, we calculated for each participant the index of asymmetry [IA = (*L* − *R*)/(*L* + *R*)]. An IA very close to zero (IA ≈ 0) will indicate equivalent durations for the dominant-right and the non-dominant-left arm movements and therefore no laterality effects. Variables showed normal distribution (Shapiro–Wilk test) and equivalent variance (Levene test). Statistical effects for the IA were tested using ANOVA with a*ge* as a between subject factor and *movement* as a within-subject factor. *Post hoc* differences assessed by means of *Scheffé* tests and the level of significance was fixed at *P* < 0.05. Moreover, the IA values in different conditions (young-actual, young-mental, elderly-actual, and elderly-mental) were compared with the value zero (0).

## Results

**Figure 2:**
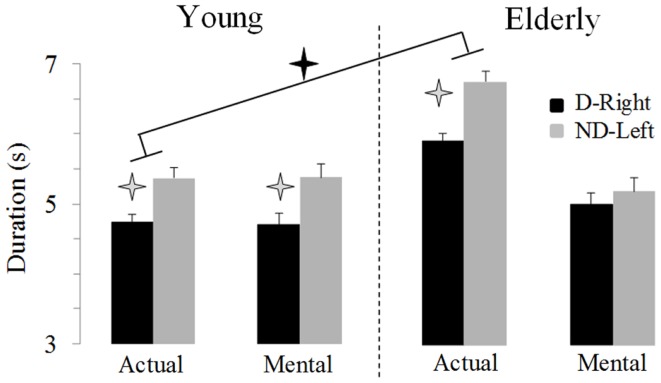
**Average durations (±SE) for the D-Right and the ND-Left arms of both groups during imagined and actual arm movements**. Asterisks (*) indicate significant differences (*P* < 0.01).

**Figure 3:**
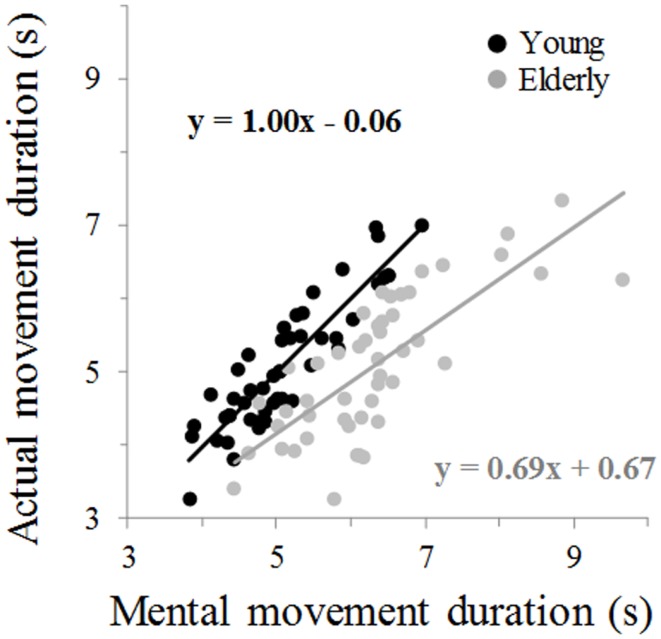
**Average mental movement duration is plotted versus the average movement duration**. The total number of marks for each group is 48 (24 participants × 2 arms). Each mark is the average value of 10 actual and 10 mental trials from one participant. The equation of the linear regression analysis is also depicted for each group.

Figure 2 illustrates average movement durations for all conditions. ANOVA showed a main effect of *age* (*F*_1,46_ = 7.8, *P* = 0.007), *arm* (*F*_1,46_ = 59.09, *P* < 0.0001), and *movement* (*F*_1,46_ = 69.25, *P* < 0.0001). Specifically, durations were shorter for the young (on average: 5.05 ± 0.16 s) than the elderly group (on average: 5.69 ± 0.19 s), shorter for the dominant-right (on average: 5.08 ± 0.15 s) than the non-dominant-left arm (on average: 5.66 ± 0.20 s), and shorter for the mental (on average: 5.06 ± 0.19 s) than the actual movements (on average: 5.68 ± 0.16 s). There was an interaction effect between *age* and *movement* (*F*_1,46_ = 66.45, *P* < 0.0001). *Post hoc* comparisons revealed that actual and mental durations significantly differed in the elderly (*P* < 0.0001), but not in the young group (*P* = 0.99). This finding is further illustrated in the Figure 3 in which the average durations of actual movements are plotted across the average durations of mental movements. The correlation between actual and mental movement durations was greater (*P* = 0.02) in the young (*r* = 0.90) compared to the elderly (*r* = 0.76) group. More appealing, however, was the significant interaction among the three factors (*F*_1,46_ = 17.16, *P* < 0.001). The *post hoc* comparison between the two groups showed significant differences for the actual right (*P* = 0.02) and the actual left (*P* = 0.004) arm movements, but not for the imagined movements (for both right and left arm, *P* > 0.1). For the young adults, *post hoc* analysis showed that actual and mental movement durations were similar (i.e., isochrony) for both arms (*P* = 0.99) and that manual asymmetries, that is greater durations for the right compared to the left arm, existed for both actual and mental movements (in both, *P* < 0.0001). For the elderly group, *post hoc* analysis showed that actual and mental movement durations differed (anisochrony) between the right and the left arm (in both, *P* < 0.0001), and that manual asymmetries existed for actual (*P* < 0.0001), but not for mental movements (*P* = 0.63).

**Figure 4:**
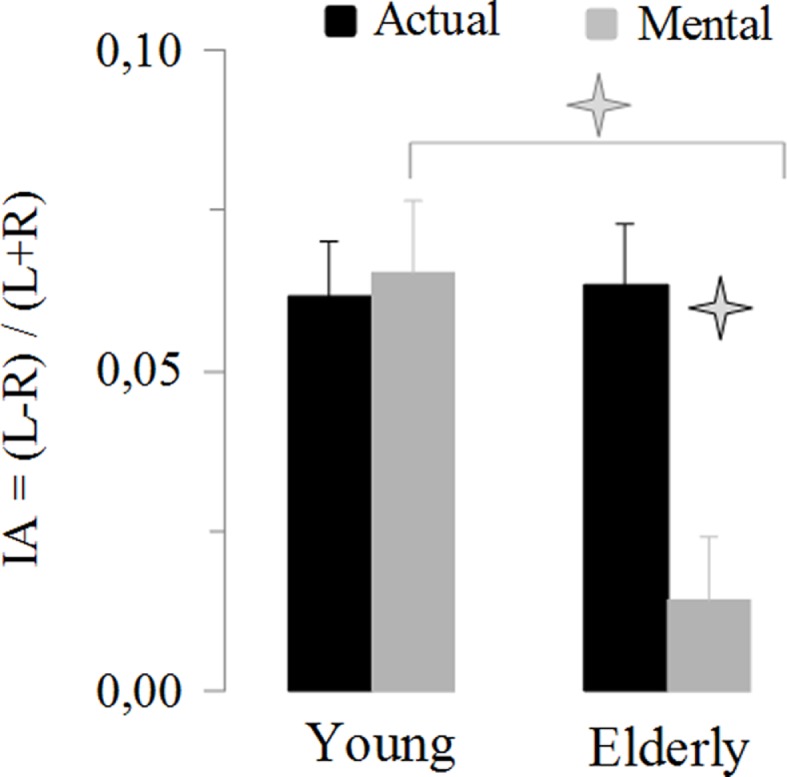
**Average values (±SE) of the index of asymmetry [IA = (*L* − *R*)/(*L* + *R*)] for the actual and mental movements in both groups**. The asterisks (*) indicate significant differences (*P* < 0.01).

This last result indicates a specific reduction in manual asymmetries in elderly adults for mental actions. To further exploit this finding, we analyzed the IA. Figure 4 shows the average values of the IA. It is noticeable that manual asymmetries for mental movements disappeared in elderly adults. ANOVA showed an interaction effect between *age* and *movement* (*F*_1,46_ = 13.14, *P* < 0.001). *Post hoc* comparisons revealed that the IA for mental movements in the elderly group was significant different (in all cases, *P* < 0.05) from all the other conditions (i.e., actual-elderly, mental-young, and actual-young). In addition, all IA values significantly differed (*t* > 5.5, df = 23, *P* < 0.0001) from the value zero (0), indicating manual asymmetry, except the IA of mental movements for the elderly group (*t* = 1.38, df = 23, *P* = 0.18).

Average RMS values are shown in Table 1. Overall, the EMG analysis showed that neither the elderly nor the young participants activated the muscles of their dominant-right or non-dominant-left arms during mental movements. Two-tailed paired *t*-test comparisons between RMS_BL_ and RMS_M_ for each muscle, arm, and group separately did not show significant differences (in all cases, *t* < 1.2, *P* > 0.3).

**Table 1:** **Root mean square (RMS) average values (±SD) from arm muscles during mental movements involving the dominant-right arm (D-right) and the non-dominant-left arm (ND-left)**.

		D-right		ND-left	
		RMS_BL_ (μV)	RMS_M_ (μV)	RMSBL (μV)	RMS_M_ (μV)
Young	DA	7 ± 2	8 ± 2	8 ± 3	9 ± 2
	BB	6 ± 3	6 ± 2	6 ± 3	7 ± 3
	DP	7 ± 3	8 ± 2	7 ± 2	8 ± 3
	TB	6 ± 2	7 ± 2	6 ± 3	7 ± 2
Eldelrly	DA	9 ± 3	9 ± 2	8 ± 2	8 ± 3
	BB	8 ± 4	7 ± 3	7 ± 2	8 ± 2
	DP	7 ± 2	7 ± 3	8 ± 3	9 ± 2
	TB	7 ± 3	8 ± 3	8 ± 2	8 ± 2

*RMS_BL_, RMS at baseline; RMS_M_, during mental movements. BB, biceps brachii; TB, triceps brachii; DA, deltoid anterior; DP, deltoid posterior*.

## Discussion

In the present study, we investigated whether right-handed aged adults preserved manual asymmetry during mental actions. With this aim, elderly and young adults carried out actual and mental pointing movements as accurate and fast as possible (speed/accuracy trade-off paradigm) with their dominant-right and the non-dominant-left arms among three targets in the horizontal plane. We recorded actual and mental movement times and compared them between arms and groups. We found that manual asymmetries (i.e., faster movements with the right arm) were preserved in young adults for both actual and mental movements. In elderly adults, manual asymmetries were observed for actual but not for mental movements (i.e., equal movement times for both arms). These findings clearly show an age-related reduction of laterality during mental actions.

### Actual movements performed with the dominant-right and the non-dominant-left arm

We found that aged participants performed arm movements significantly slower than young participants. This result expands those of previous studies, which have reported a pronounced increase in movement time with age in a variety of tasks. Due to dedifferentiation of cognitive and motor neural resources, a decrease in processing speed acts as a common cause to behavioral slowing in both cognitive and motor tasks (Sleimen-Malkoun et al., 2013; Temprado et al., 2013). This slowing in movement speed may indicate a strategic adaptation: older adults emphasize movement accuracy at the cost of movement speed (Seidler-Dobrin and Stelmach, 1998; Seidler-Dobrin et al., 1998). Slower information processing may also affect motor performance in a non-specific way due to an increase in neural noise (Salthouse and Somberg, 1982a,b; Salthouse, 1993; Salthouse and Coon, 1993). In general, elderly adults, compared to young adults, show lengthened deceleration curves, lower peak velocities, and secondary corrective sub-movements during movements involving the right arm (Bellgrove et al., 1998; Seidler-Dobrin and Stelmach, 1998; Seidler-Dobrin et al., 1998; Ketcham et al., 2002). We also observed that actual movements involving the dominant-right arm were significantly faster than actual movements involving the non-dominant-left arm in both age groups. This result corroborates those of previous investigations involving young adults, which have also reported better temporal performances for the right compared to the left arm in reaching tasks requiring spatial (Maruff et al., 1999a; Personnier et al., 2008; Skoura et al., 2008) and/or dynamic (Sainburg and Kalakanis, 2000; Bagesteiro and Sainburg, 2002) constraints. Additionally, our findings are in line with those of previous studies which have shown that manual asymmetries in reaction and movement time do not change with age (Francis and Spirduso, 2000) and could even increase (Mitrushina et al., 1995). However, recent experimental evidence has shown that kinematic variables, such as path curvature and final position error, are asymmetric between the dominant-right and the non-dominant-left arm in young adults, but symmetric in older adults (Przybyla et al., 2011). Similarly, it has been proposed an aged-related attenuation of dominant hand superiority in fine arm movements (Kalisch et al., 2006). As there is increasing evidence for differential manifestation of handedness in brain function and behavior in older adults (Bernard and Seidler, 2012), more investigations are needed to better understand the interactive effects of age and handedness.

### Mental movements performed with the dominant-right and the non-dominant-left arm

Our findings showed that laterality was preserved in the young group during mental actions. This observation is added to those of previous studies which have reported that lateralization emerges in covert states of action (Maruff et al., 1999a; Skoura et al., 2008, 2009). The most interesting finding in the present study was that laterality completely disappeared in the elderly group during mental actions. This finding seems to be consistent with the *HAROLD* model, which proposes that frontal activity during cognitive performance tends to be less lateralized in older than in younger adults (Cabeza, 2002). Likewise, recent studies have shown that hemispheric recruitment becomes more symmetric (Reuter-Lorenz and Lustig, 2005; Seidler et al., 2010) and manual asymmetry decreases with age (Kalisch et al., 2006; Przybyla et al., 2011; Wang et al., 2011). This attenuation in brain lateralization during cognitive tasks in elderly people may also explain why laterality disappears during mental movements. The strong reduction in manual asymmetry during mental actions in elderly adults may also have functional origins related to the concept of use-dependent plasticity. The advantage of the dominant arm is determined early in life and is strengthened by practice through everyday activities. When these activities decrease in latter life (Hughes et al., 1997; Schut, 1998; Ranganathan et al., 2001) it is conceivable that the practice-based superior performance of the dominant-right arm is no longer maintained, thus approaching the performance level of the non-dominant-left arm. This is in line with the more balanced use of both arms in everyday life of aged subjects (Carmeli et al., 2003). Our findings seem to not confirm the *right hemi-aging hypothesis*, which postulates that age-related cognitive decline affects functions attributed to the right hemisphere to a greater degree than those associated with the left hemisphere (Brown and Jaffe, 1975). As mental movements are lateralized in the left motor cortex of right-handers (Fadiga et al., 1999; Sabate et al., 2004; Stinear et al., 2006) one could expect an increase in the right arm dominance in later life, but this was not the case.

It is of interest in our study that reduction of laterality in elderly adults is observed for mental but not for actual movements. We can speculate that this might be due to weakness in internal forward model prediction that occurs with an increase in age. Indeed, we found a discrepancy between imagined movement time (estimated state) and actual movement time (actual state) in elderly adults for both arms, indicating imprecise predictions from the internal forward model. During actual movements laterality is conserved possibly via feedback-related mechanisms that update state estimation and performance. During mental movements, sensory information is lacking because no movement occurs and state estimation is not updated, reflecting thus similar action representations for both arms. This original finding may suggest a progressive decline in action planning, affecting mental states of action earlier than motor states. In such a case, mental movement simulation may be an interesting tool to detect early declines in motor planning. The bilateral alteration of the temporal processing of imagined actions in elderly adults could be attributed to a failure in the recruitment of cortical motor areas involved in mental movement simulation, but it is more-likely due to an over-activation of brain areas, which may reflect neural compensation mechanisms in the aging brain (Ward and Frackowiak, 2003; Heuninckx et al., 2005, 2008; Nedelko et al., 2010; Zwergal et al., 2010; Saimpont et al., 2013). The lack of corticospinal selectivity during imagined actions, revealed by transcranial magnetic stimulation experiments (Leonard and Tremblay, 2007), further supports a cortical origin of this age-related decline in motor imagery ability. Addition, age-related differences in mental movements could be partially attributed to anatomical changes in the corpus callosum. Recent studies have demonstrated that elderly had significantly smaller callosal area in the anterior and mid-body of the corpus callosum than young adults (Fling et al., 2011). These structural alterations cause changes in interhemispheric communication such that older adults may rely on the bilateral cortical cooperation to a greater extent than young adults for unimanual and bimanual mental motor tasks (Fling and Seidler, 2012). Further studies, investigating both unimaunal and bimanual mental movements in elderly could provide valuable information regarding interhemispheric control in metal movements (Garbarini et al., 2013; Piedimonte et al., 2013).

### EMG patterns during mental movements

Current results cannot be ascribed to a muscle activation strategy because the analysis of the EMG patterns showed that both young and aged participants did not activate the muscles of their dominant-right or non-dominant-left arm during motor imagery. These results expand those of previous investigations, which have shown that muscles normally involved in the execution of an action remained silent when subjects mentally simulated the same action (Hashimoto and Rothwell, 1999; Gentili et al., 2006; Personnier et al., 2008, 2010a,b; Papaxanthis et al., 2012). Note, however, that muscle inactivation during imagined arm movements was not the only possible outcome in the present study. Indeed, previous investigations have shown that inhibition of descending volleys was not always complete during imagined actions (Guillot and Collet, 2005; Lebon et al., 2008). Therefore, we cannot exclude the possibility that muscle activation above the BL could occur in other muscles during other motor tasks. Note, that motor overflow in elderly adults is more frequently observed in finger muscles (Hoy et al., 2004; Addamo et al., 2007).

## Conclusion

In synopsis, our results indicate similar temporal performance between the left and the right arm in elderly adults during imagined movements. However, whether these behavioral findings are related to systemic changes in neural recruitment during mental tasks cannot be determined by the current data, and must await further research. Furthermore, our experiment involved arm pointing movements. Whether reduction in manual asymmetry during mental movements can be generalized to other motor tasks, implying dynamic constraints and/or interaction with objects, has to be verified. Our results may have important clinical implications as, through the motor imagery process, therapists may have indirect access to motor representations and possibly detect impairments at the level of action planning. Temporal discrepancies between actual and mental movements may be a useful indicator to test whether an action is executed as planned (imagined movement).

## Conflict of Interest Statement

The authors declare that the research was conducted in the absence of any commercial or financial relationships that could be construed as a potential conflict of interest.
